# Transgenic *Arabidopsis* Plants Expressing Tomato Glutathione S-Transferase Showed Enhanced Resistance to Salt and Drought Stress

**DOI:** 10.1371/journal.pone.0136960

**Published:** 2015-09-01

**Authors:** Jing Xu, Xiao-Juan Xing, Yong-Sheng Tian, Ri-He Peng, Yong Xue, Wei Zhao, Quan-Hong Yao

**Affiliations:** Biotechnology Research Institute, Shanghai Academy of Agricultural Sciences, Shanghai, 201106, China; Texas Tech University, UNITED STATES

## Abstract

Although glutathione S-transferases (GST, EC 2.5.1.18) are involved in response to abiotic stress, limited information is available regarding gene function in tomato. In this study, a GST gene from tomato, designated *LeGSTU2*, was cloned and functionally characterized. Expression profile analysis results showed that it was expressed in roots and flowers, and the transcription was induced by salt, osmotic, and heat stress. The gene was then introduced to *Arabidopsis* by *Agrobacterium tumefaciens*-mediated transformation. Transgenic *Arabidopsis* plants were normal in terms of growth and maturity compared with wild-type plants. Transgenic plants also showed an enhanced resistance to salt and osmotic stress induced by NaCl and mannitol. The increased tolerance of transgenic plants was correlated with the changes in proline, malondialdehyde and antioxidative emzymes activities. Our results indicated that the gene from tomato plays a positive role in improving tolerance to salinity and drought stresses in *Arabidopsis*.

## Introduction

Environmental stress factors, such as drought and salinity, affect plant growth and development by causing osmotic stress and water deficit [1]. Plants develop various biochemical and physiological mechanisms to respond and adapt to drought and salt stresses. One of these mechanism is the expression of some stress-inducible genes (e.g. enzymes involved in the biosynthesis of various osmoprotectants) to counteract the detrimental conditions [2–3].

Glutathione S-transferases (GSTs; EC 2.5.1.18) are a well-characterized detoxification enzyme family involved in stress tolerance, which catalyze the conjugation of reduced tripeptide glutathione (GSH) to electrophilic substrates. GSTs were first discovered because these enzymes can metabolize various toxic exogenous compounds (xenobiotics) by GSH conjugation [4]. Plant GSTs are commonly known for their role in herbicide detoxification. Besides, plant GSTs are also considered as glutathione peroxidases that directly detoxify electrophiles. Furthermore, GSTs function as non-enzymatic carriers (ligandins) in intracellular transport and catalyze anthocyanin-GSH conjugates, thereby allowing transport into vacuoles via a glutathione pump [5]. Moreover, several plant GSTs exhibit peroxidase activity and may play roles in enhancing tolerance to chilling, osmotic dehydration, and herbicide-induced damage [6–8].

Plants GSTs are classified into eight classes: phi, tau, theta, zeta, lambda, glutathione-dependent dehydroascorbate reductases (DHARs), tetrachlorohydroquinone dehalogenase (TCHQD) and membrane associated proteins in eicosanoid and glutathione metabolism (MAPEG). Among these eight classes, phi and tau are the largest plant-specific and often highly stress-inducible GSTs [9]. The role of GSTs in stress has been demonstrated in several transgenic studies, and over-expression of GSTs in tobacco [8] and rice [10] increased transgenic plant capability to endure harsh treatments such as high and low temperatures and salt concentration. Kumar et al. showed that over-expressing of *OsGSTL2* enhanced the tolerance to heavy metals and other abiotic stresses like cold, osmotic and salt in GST transgenic plants [11]. Moreover, expression of *ThGSTZ1* from *Tamarix lhispida* in *Arabidopsis* can improve drought and salinity tolerance in transgenic plants [12]. Transgenic tobacco over-expressing cotton GST showed enhanced resistance to methy1 viologen [13]. A recent study on *AtGSTU17* showed that it plays a negative role in drought and salt stress tolerance. When *AtGSTU17* was mutated, plants were more tolerant to drought and salt stresses compared with wild-type plants [14].

Although many GST genes have been cloned from *Arabidopsis* [14–15], rice [16], tobacco [17], soybean [18], maize [19], poplar [20], sorghum [21] and other plants, the role of a GST gene from tomato has been rarely investigated. In this study, a *GST* coding sequence names *LeGSTU2* from tomato (*Lycopersicon esculentum*) was cloned by PCR method and was functionally characterized by heterologous expression in *Arabidopsis*. Detailed analysis carried out on transgenic lines developed in this study suggests the role of *LeGSTU2* in abiotic stresses tolerance, particularly in osmotic and salt stress conditions.

## Materials and Methods

### Plant materials, growth conditions, and treatments

Plants (*Arabidopsis thaliana* ecotype Columbia L.) were grown in a growth chamber at 22°C on MS medium or in pots filled with vermiculite/peat moss/perlite (9:3:1) mixture. The plants were kept in a 16/8h day/night cycle at a light intensity of ~120μmol photons m^-2^ s^-1^.

Tomato seeds (*Lycopersicon esculentum*) were sterilized with 75% (v/v) ethanol for 5 min, followed by commercial bleach (0.5% sodium hypochlorite) for 20min, then rinsed thrice with sterile deionized water and sown onto plates containing MS with 1% agar and 4% (w/v) sucrose. The plates were placed in the dark for 3–4d. After the seeds germinated, the plates were transferred to a controlled environment chamber at 22°C and subjected to a 16/8 h day/night cycle. After 7 d, the seedlings were transplanted to pots (vermiculite/peat moss/perlite mixture, 9:3:1). Three-week-old tomato seedlings were watered with 200mM NaCl, 50mM mannitol, 4°C (cold) or 40°C (heat) for 0, 1, 3, 6, 12, and 24h, respectively. The whole plants at each treatment or the different organs (roots, stems, leaves, flowers or fruits) from untreated plants were pooled, frozen in liquid N_2,_ and stored at -70°C.

### Total RNA extraction and reverse transcription

Total RNA was isolated using a Multisource Total RNA Miniprep kit (Axygen Scientific, CA, USA) according to the manufacturer’s instructions. First-strand cDNA synthesis was conducted with 5 μg of total RNA by using a TransScript Fly First-strand cDNA Synthesis SuperMix (Transgen Biotech, Shanghai, China).

### Cloning and sequence analysis of *LeGSTU2*

The *LeGSTU2* (GenBank accession number: AY082341) coding sequence was cloned from tomato (*L*.*esculentum*) cDNA using PCR method with specific primers for *LeGSTU2* genes, forward: *LeGSTF2*: 5'-AAGGATCCATGGCTAATGATCAGGTG-3' and reverse: *LeGSTZ2*: 5'-AAGAGCTCTTATTCAAGTCCAAG-3'). Clones containing the *LeGSTU2* coding sequence were further sequenced from both sides to confirm their sequences. Molecular weight (MW) and isoelectric point (pI) predictions for the deduced protein were performed using the Compute pI/Mw tool (http://web.expasy.org/protparam/). Phylogenitic tree construction was performed using the Clustal W2 program.

### Real-time PCR

Tomato seedlings from different treatments (as described in the previous section) were harvested to analyze *LeGSTU2* expression profile. Total RNA was isolation and reverse-transcribed as described in a previous section. PCR amplification was performed with specific primers for *LeGSTU2* genes, forward: *LeGSTF1*: 5'-GGGAGACGAACAAGAGGC-3' and reverse: *LeGSTZ1*: 5'-CCACAAGGTTTGGGCACT-3'. Amplification of tomato *Actin* gene (GenBank accession number: BT012695) was used as an internal control [22]. Primers as following: forward: *LeAcF*: 5'-TGAAATGTGACGTGGATATTAGG-3' and reverse: *LeAcZ*: 5'-TGAGGGAAGCCAAGATAGAGC-3'.

Arabidopsis plants of three-week-old were harvested to analyze the positive transformants. Specific primers information for *LeGSTU2* was mentioned in the previous part. *A*. *thaliana actin* gene (*AtActin2*, GenBank Accession number: U41998) was used as an internal control, primers as following: forward: AtAc2F: 5'-AGTAAGGTCACGTCCAGCAAGG-3' and reverse: AtAc2Z: 5'-GCACCCTGTTCTTCTTACCGAG-3'.

The cDNA was amplified using SYBR Premix Ex Taq (TaKaRa, Shanghai, China) as described [23]. The PCR program was 94°C, 1min, 35 cycles of 94°C, 20s; 54°C, 20s; 72°C, 20s, 81°C for 1s for plate reading. Each sample reaction was carried out in triplicate to ensure the reproducibility of the results.

### Vector construction and *Arabidopsis* transformation

The complete ORF of *LeGSTU2* was amplified by PCR using gene-specific primers as described, cloned in TA clone vector Simple pMD-18 (Takara) and sequenced. The fragment was then cloned in the plant expression vector pYK4102 [23] at *Bam*HI and *Sac*I sites under the control of CaMV35S promoter. The construct was mobilized into *A*. *tumefaciens* GV3101 and transformed in plants to generate GST transgenic lines of *Arabidopsis* by using a floral dip method [24]. Positive transformants in hygromycin (50μg/ml) plates were selected and confirmed by PCR using the same primers. T3 generation was selected for further experiments

### GST activity measurement

GST activity was determined according to Ji et al. [25] with minor modification. Fresh leaf tissues (~ 0.1 g) were obtained from three-week-old plants grown under normal conditions. The leaves were harvested and homogenized in 1.5 ml of chilled 50 mM phosphate buffer by using a chilled pestle and mortar. The homogenate was centrifuged at 20,000g for 10 min in a refrigerated centrifuge at 4°C. The supernatant was stored at 4°C and used for enzyme assays within 4 h. Enzyme activity was evaluated in the presence of 1mM glutathione in 100mM sodium phosphate buffer (pH 6.5). The reaction was initiated by adding 1-chloro-2, 4-dinitrobenzene (CDNB) to a final concentration of 1mM. Changes in A340 were measured and background levels of spontaneous CDNB decay were subtracted.

### Analysis of stress tolerance in transgenic lines

Stress tolerance was analyzed using three homozygous transgenic lines (OE3, OE7 and OE10) expressing *LeGSTU2* in T3 *Arabidopsis* generation. To evaluate the effect of abiotic stresses on WT and transgenic plants, seeds were germinated on MS media containing NaCl (150 mM and 200 mM) and mannitol (150 mM and 300 mM). Seed germination was subsequently monitored and recorded at an interval of 12h during growth. Root length was recorded after 10 d of germination.

To evaluate salt/drought stress tolerance, three-week-old plants were watered with NaCl (250 mM) as salt stress, or withheld watering for two weeks as water deficit stress. Then the plants were irrigated again and the performances of WT and transgenic plants were compared.

To measure the physiological parameters involved in stress tolerance, three-week-old plants were treated with 250mM NaCl (as salt stress) or 300 mM Mannitol (as osmotic stress) for 5d, and the proline content, MDA content, chlorophyll content, SOD and POD activity were measured according to the study of Diao et al. [26]. Proline was assayed on water-extracted seedlings using the ninhydrin assay. The malondialdehyde (MDA) content was determined by the reaction of thiobarbituric acid (TBA). Superoxide dismutase (SOD, EC 1.15.1.1) activity was determined by monitoring the inhibition of photochemical reduction of nitro blue tetrazolium.

Aerial parts from three-week-old plants were excised to analyze the standardized water content. The loss in fresh weight was monitored at indicated times. Detached aerial parts were then dried at 80°C for 5h to determine dry weight. It was calculated as [(FW_*i*_-DW)/(FW_0_-DW)]×100, where FW_0_ and FW_*i*_ are fresh weight for original and any given internal fresh weight, respectively, and DW is dry weight. These tests were conducted in the controlled environment chamber at 22°C.

### Statistical analysis

ANOVA was performed using Duncan’s multiple comparison tests. Statistically significant differences (*P* < 0.05) are reported in the text and shown in the figures.

## Results

### Cloning and characterization of *LeGSTU2*

**Fig 1:**
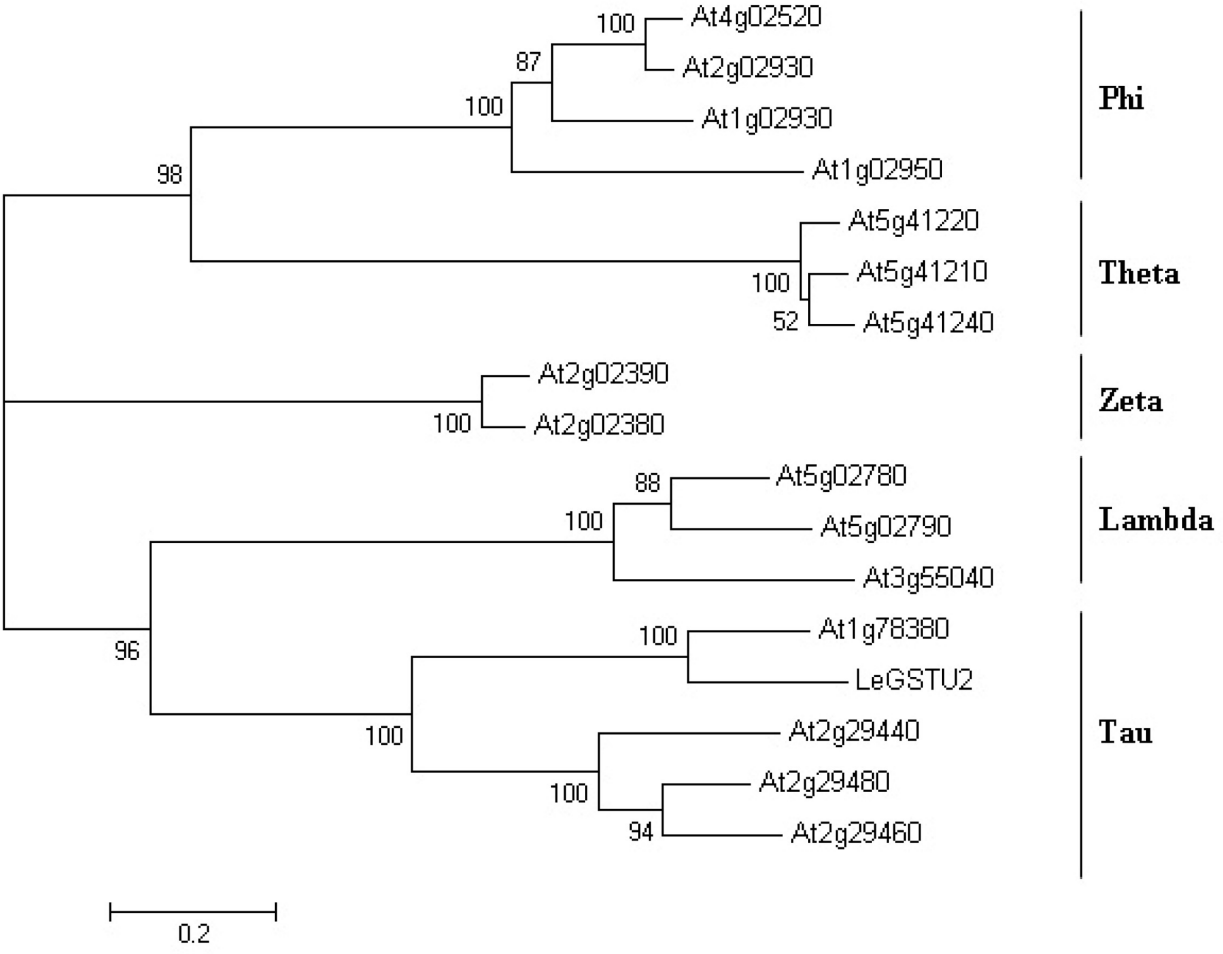
Phylogenetic tree for the amino acid sequences of LeGSTU2 with the GST proteins from *Arabidopsis thaliana*. The NJ method was applied using Mega 4 from a tree file produced by Clustal W2.

The coding region of *LeGSTU2* was 663 bp in length and was predicted to encode a 220 amino acid protein with a calculated MW of 25.4 kDa and a pI of 5.51. Multiple sequence alignments showed that LeGSTU2 share the highest similarity with AtGSTU19 (At1g78380), AtGSTU6 (At2g29440), AtGSTU2 (At2g29480) and AtGSTU4 (At2g29460), which suggests that *LeGSTU2* may be a lambda-type GST (Fig 1).

### Expression of *LeGSTU2* transcripts

**Fig 2:**
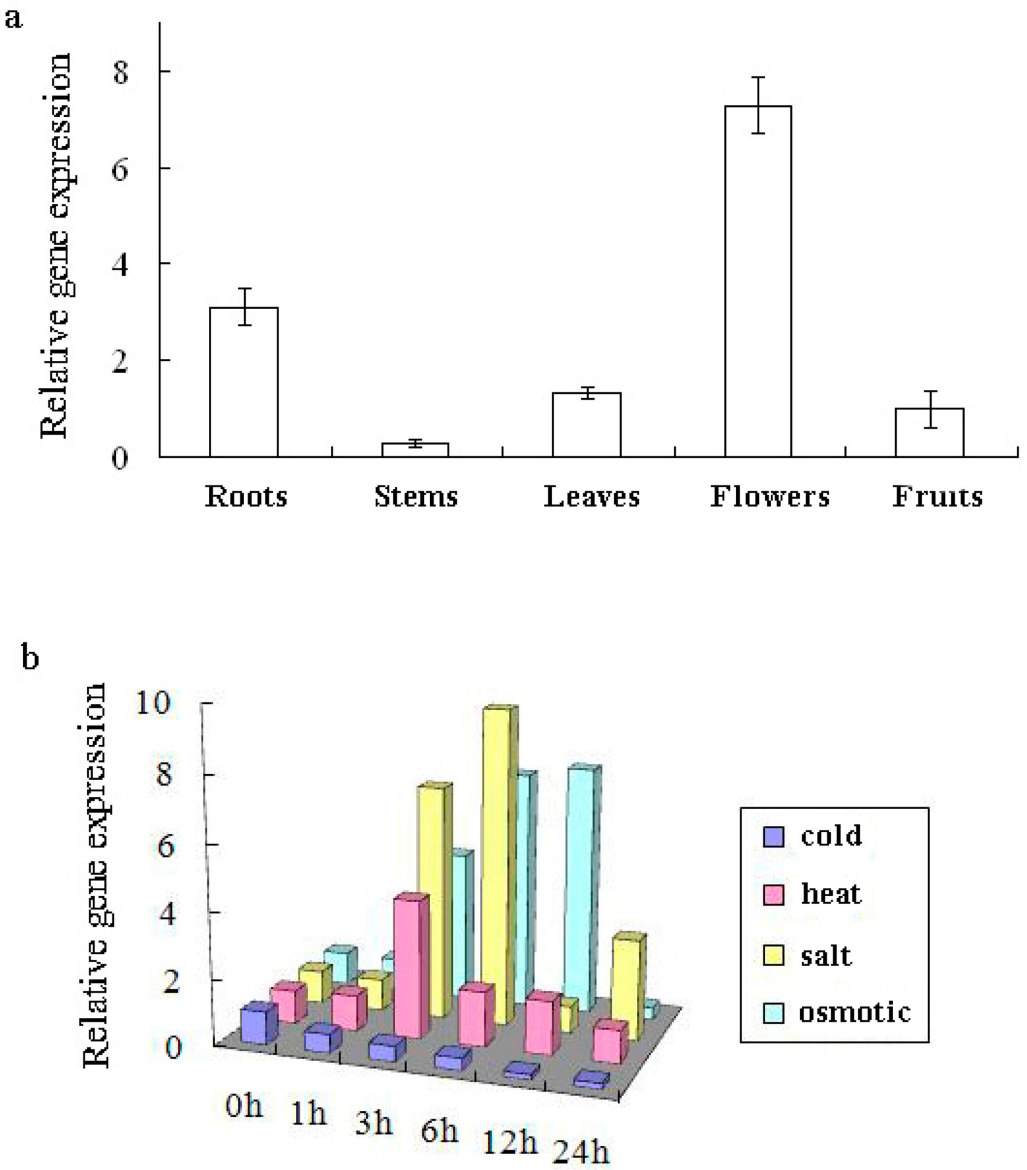
Expression profile of *LeGSTU2* in tomato. a, The expression of *LeGSTU2* in different organs by real-time PCR. Total RNA was isolated from different organization (roots, stems, leaves, flowers or fruits) of tomato seedlings. The experiment was replicated three times and the mean value is shown. b, Expression of *LeGSTU2* transcripts in response to drought, salt, cold and heat stresses by real-time PCR. Total RNA was isolated from tomato seedlings, which was exposed to different stress at indicated times (0 h as a control). 200 mM NaCl for salt stress, 50mM Mannitol for osmotic stress, 40°C for heat stress and 4°C for cold stress. The experiment was replicated three times and the mean value is shown.

Different organs of tomato seedlings were harvested, and the expression of *LeGSTU2* was analyzed by real-time PCR to examine the expression profile. The results showed that *LeGSTU2* was highly expressed in roots and flowers, suggesting that *LeGSTU2* could participate in root growth and flower development (Fig 2A).

The expression of *LeGSTU2* under stress conditions was determined by Real-Time PCR. The results showed similar patterns in response to salt, osmotic and heat treatment. *LeGSTU2* transcripts were detected in 3h and the highest transcript level was reached at 6 h after salt or osmotic treatment was initially administered (Fig 2B). Under heat stress, *LeGSTU2* transcripts were analyzed and the highest level was reached at 3h. These observations suggested that *LeGSTU2* was induced by salt, osmotic and heat stress. However, *LeGSTU2* expression was repressed by low temperature.

### Expression of *LeGSTU2* in *Arabidopsis*

**Fig 3:**
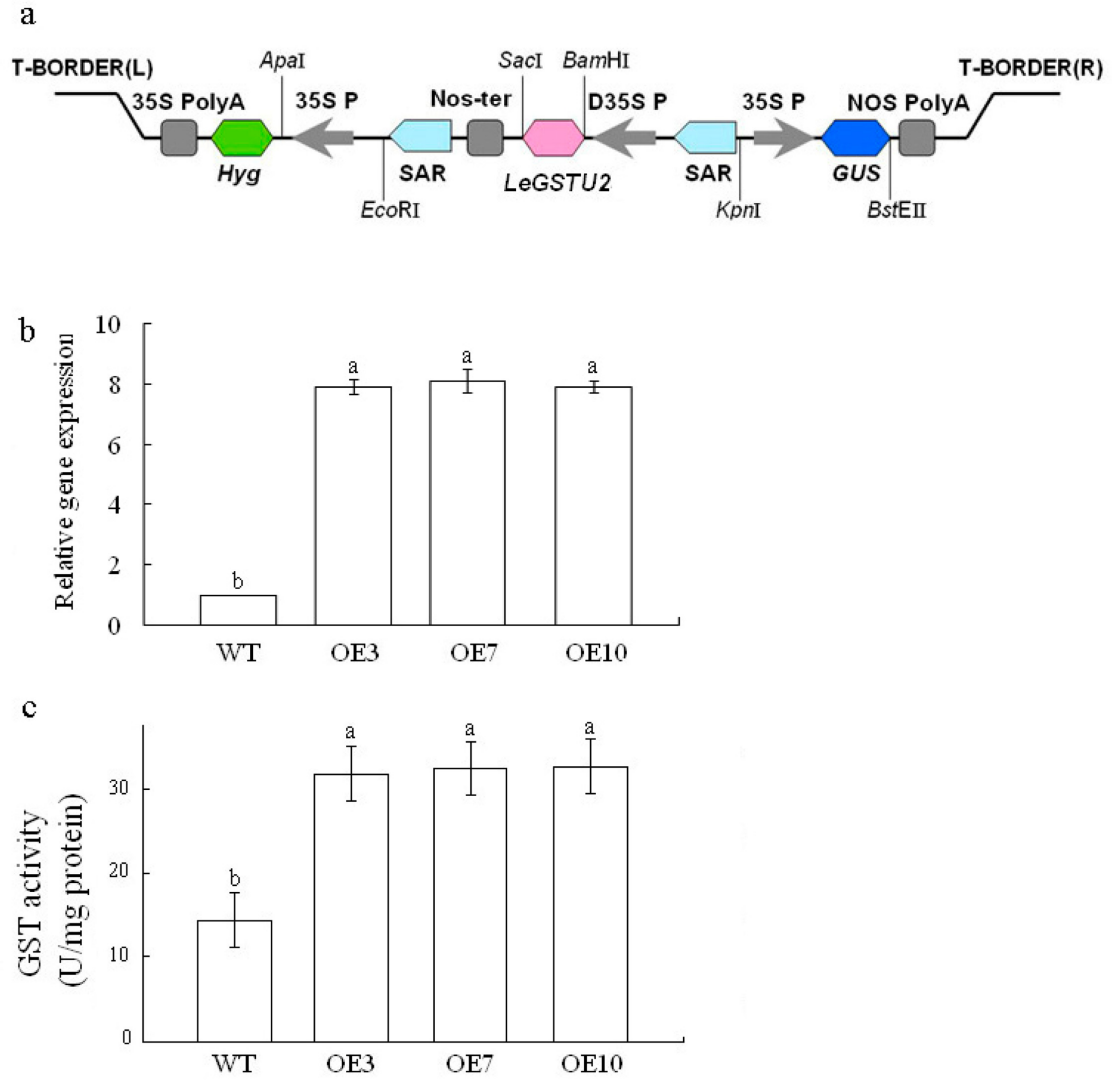
Transgenic *Arabidopsis* plants with *LeGSTU2*. a, The *LeGSTU2* expression vector for *Arabidopsis* transformation. *Nos-Ter* terminator sequence of the nopaline synthase, *SAR* scaffold attachment region. b, Expression of *LeGSTU2* in the three transgenic lines and in the wild-type. Total RNA was isolated from three-week-old plants grown under normal conditions. Values are means ± SD (n = 3) and different letters above bars indicate significant differneces (P < 0.05) among different lines. c, GST activity in *Arabidopsis* plants expressing LeGSTU2. GST activity was measured in leaves of three-week-old plants growth under normal condition using CDNB as substrate. Values are means ± SD (n = 3) and different letters above bars indicate significant differneces (P < 0.05) among different lines.

*LeGSTU2* cDNA was cloned into plant expression vector pYK4102 (Fig 3A) and introduced to *Arabidopsis* cells by using *A*. *tumefaciens*-mediated floral dip method. Ten independent lines of transgenic plants (T1 generation) were generated. Among these lines, three homozygous transgenic lines (named OE3, OE7 and OE10) expressing *LeGSTU2* of T3 generation were selected for further experiments.

RT-PCR analysis results confirmed that *LeGSTU2* was detected in the three transgenic lines in the T3 generation, and no amplification was observed in WT (Fig 3B). GST activity levels were analyzed in both transgenic plants and WT. Compared with WT, different transgenic lines showed a significantly enhanced GST activity when CDNB was used as substrate (Fig 3C). No obvious effects on growth and development were observed in *LeGSTU2* transgenic plants under normal growth conditions.

### Response of transgenic lines to salt stress and osmotic stress

**Fig 4:**
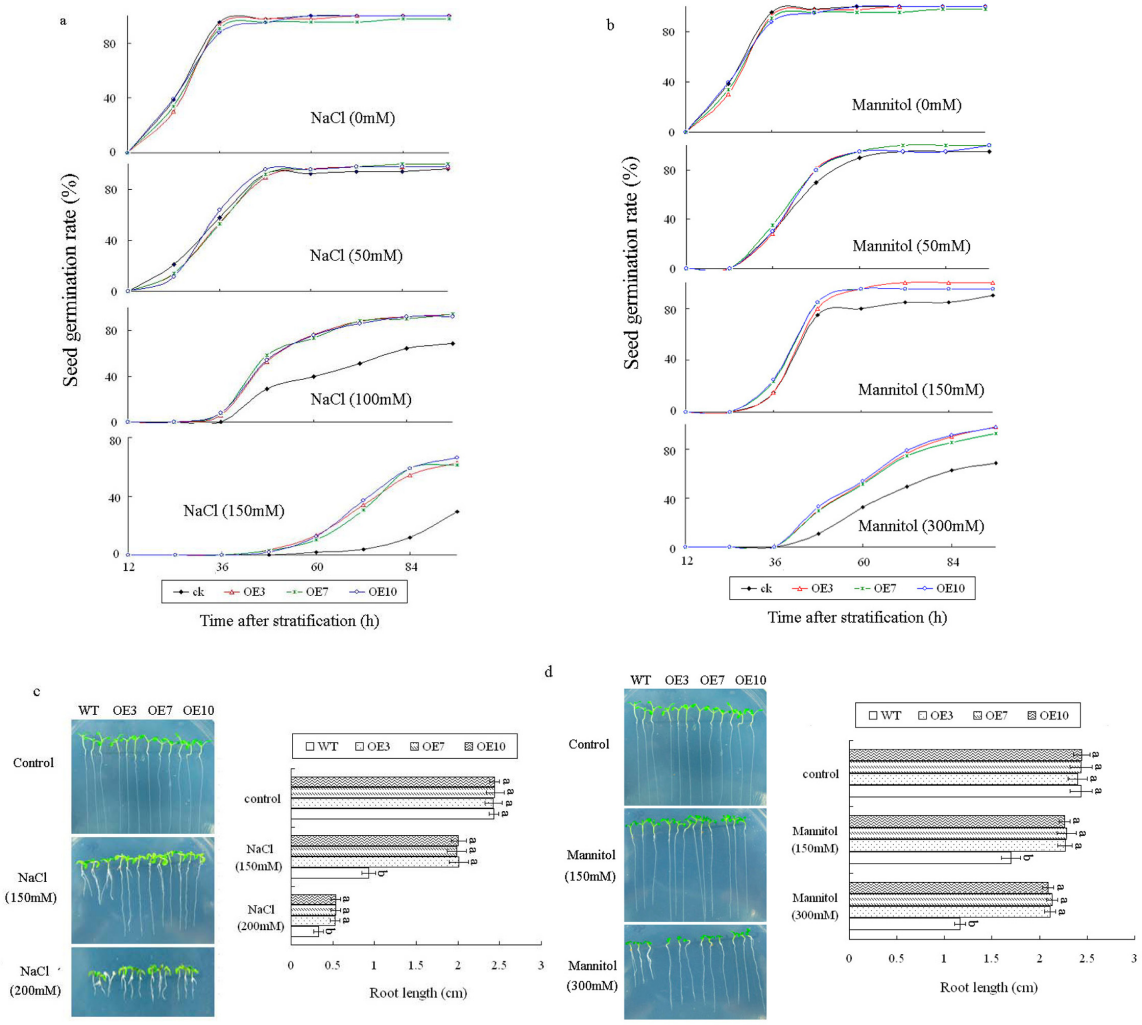
Response of *LeGSTU2* transgenic plants to salt and osmotic stress. a,b, Seed germination. The germination rates were recorded in MS medium supplemented with different concentration of NaCl (as salt stress) and mannitol (as osmotic stress) during a period from 12 to 96 h after stratification. Each value is the mean ± SD (n = 3) of at least 50 seeds. c,d, Phenotypes and primary root length of different genotype under salt stress (c) and osmotic stress (d). Seeds were germinated on MS medium containing different concentrations of NaCl as salt stress and mannitol as osmotic stress. Photograph was taken after 10 days of germination. Each value is the mean ± SD of at least 50 seedlings. Different letters above bars indicate significant differences (P < 0.05) among different genotypes in the same treatment.

Transgenic *Arabidopsis* lines over-expressing *LeGSTU2* gene were germinated in medium containing different concentrations of NaCl (150 and 200mM) as salt stress or Mannitol (150 and 300mM) as osmotic stress to analyze the role of *LeGSTU2* under stress. There was no significant difference in the germination rates and root length between WT and transgenic plants under normal growth conditions. However, the germination rate and root length were increased in the transgenic lines compared with WT under salt and osmotic stress conditions (Fig 4). Under salt stress, the average germination rates of transgenic seedlings were 93.5%, 63.4%, compared with 68.9%, 29.4% of the WT in the medium (96h) containing 100 and 150 mM NaCl, respectively (Fig 4A). Under 300 mM mannitol stress conditions, the germination rate of WT was 68.6%, while the average germination rate of transgenic plants was 96.5% (Fig 4B).

The growth of both transgenic plants and WT was significantly repressed by both salt and osmotic stress, but the growth of WT was obviously decreased compared to that of the transgenic lines. The average root lengths of the transgenic lines were 2.2-, 1.7-fold more than the WT under 150mM, 200mM NaCl treatments, respectively (Fig 4C). Likewise, the average root lengths of the transgenic lines were 1.3-, 1.8-fold longer than WT under 150mM, 300mM mannitol, respectively (Fig 4D). These results suggested that transgenic plants enhanced tolerance to salt and osmotic stress.

### Stress tolerance of transgenic plants

**Fig 5:**
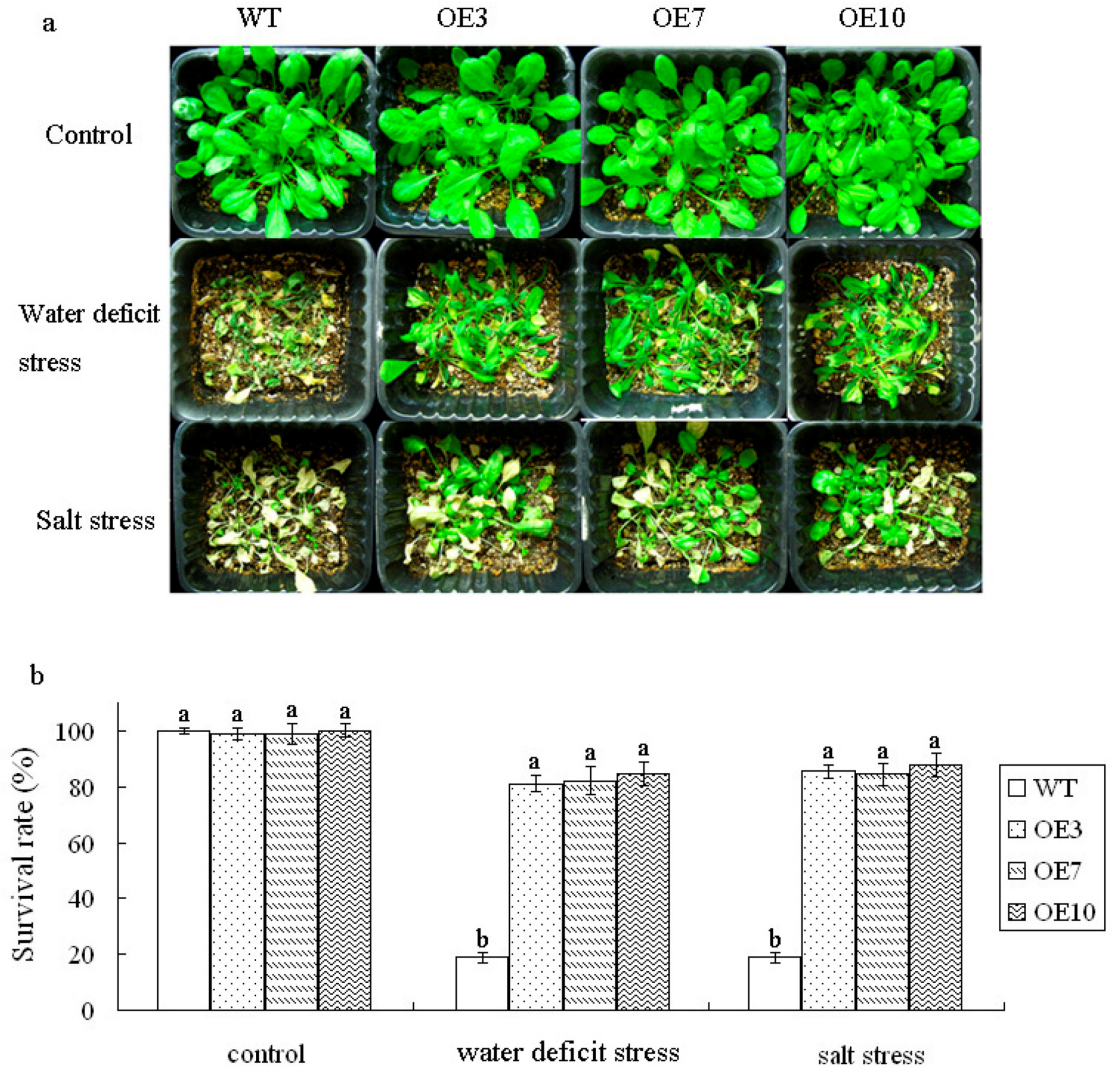
Stress tolerance of transgenic plants. a Three-week-old plants with different transgenic lines (OE3, OE7 and OE10) were given salt and water deficit stress treatment. Control, 3-week-old plants growing under normal conditions. Salt stress, plants watered with NaCl (250 mM). Water deficit stress, plants withheld watering for 2 weeks, then irrigated again. The experiment was replicated three times with similar results and for 48 plants per line for each experiment. b, Survival rate of different lines under stress treatment mentioned in a. Values are mean±SD of three individual experiments. The statistical significance was done and determined by Duncan’s multiple comparison tests. Different letters above bars indicate significant differences (P < 0.05) among different genotypes in the same treatment.

Three-week-old seedlings were watered with NaCl (250 mM) for salt stress, or with withheld watering for water deficit stress to further analyze salt/drought tolerance. After two weeks, some plants exhibited lethal effects, then the plants were irrigated again. The plants were photographed at 15 d after these plants were exposed to stress, the survival rate was then measured (Fig 5). Under salt stress condition, >80% of the transgenic plants survived, whereas only 20% of WT survived. Under water deficit stress for two weeks, >80% of the transgenic plants survived, whereas approximately 20% of WT plants survived.

**Fig 6:**
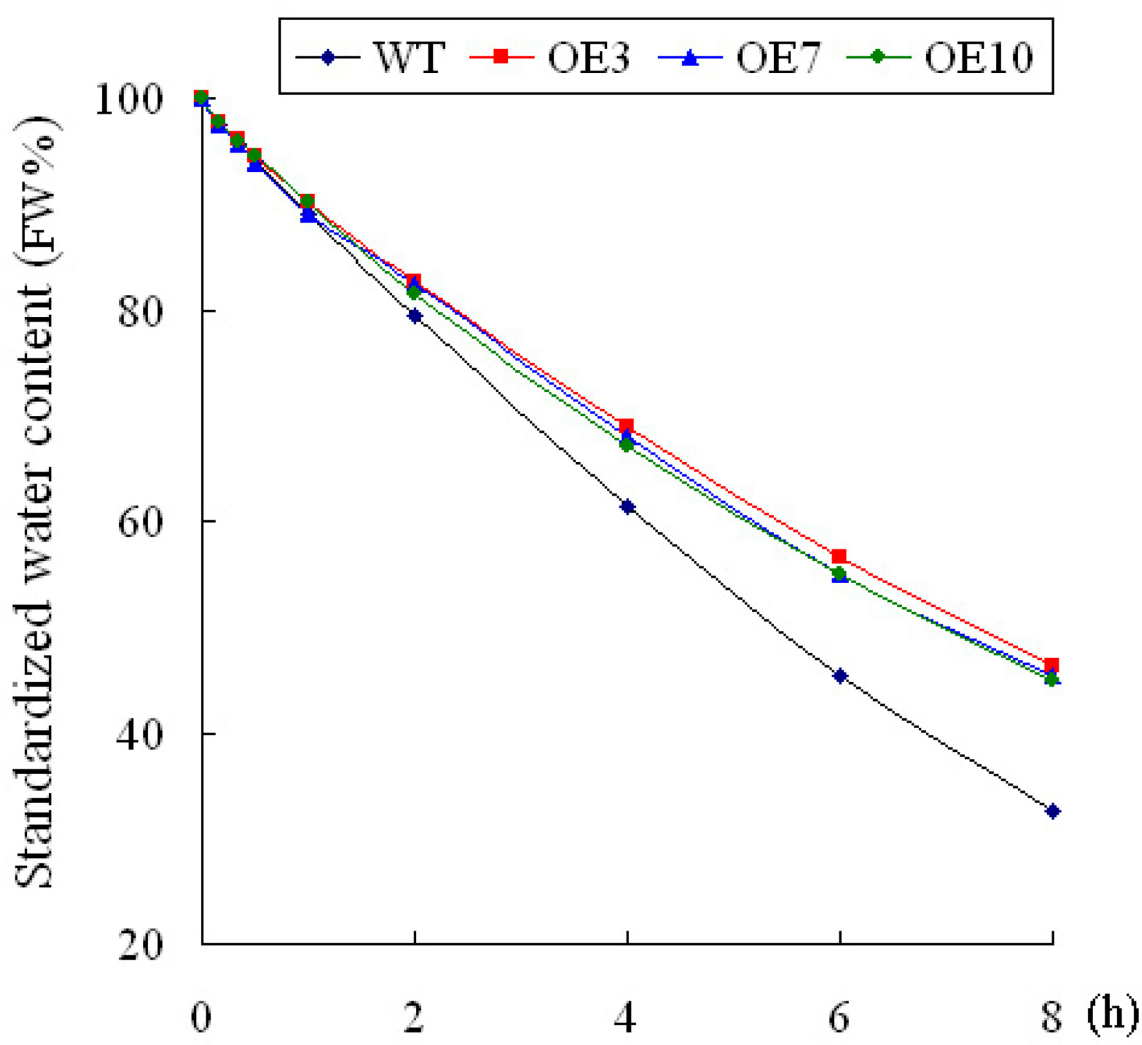
Standardized water content of different lines. Each data point is the mean ± SD of three replicates, each from ≥10 plants.

The water content was analyzed further. When the leaves of WT and transgenic plants were exposed to air for 2h, the average water content rate of the transgenic plants was 82.3%, whereas the rate was 79.3% in WT. At 8h, the average water content rate of transgenic plants was 45.7%, which was 1.4-fold higher than that of WT. These results indicated that transgenic plants presented a higher capacity to conserve water than those of WT plants (Fig 6).

**Fig 7:**
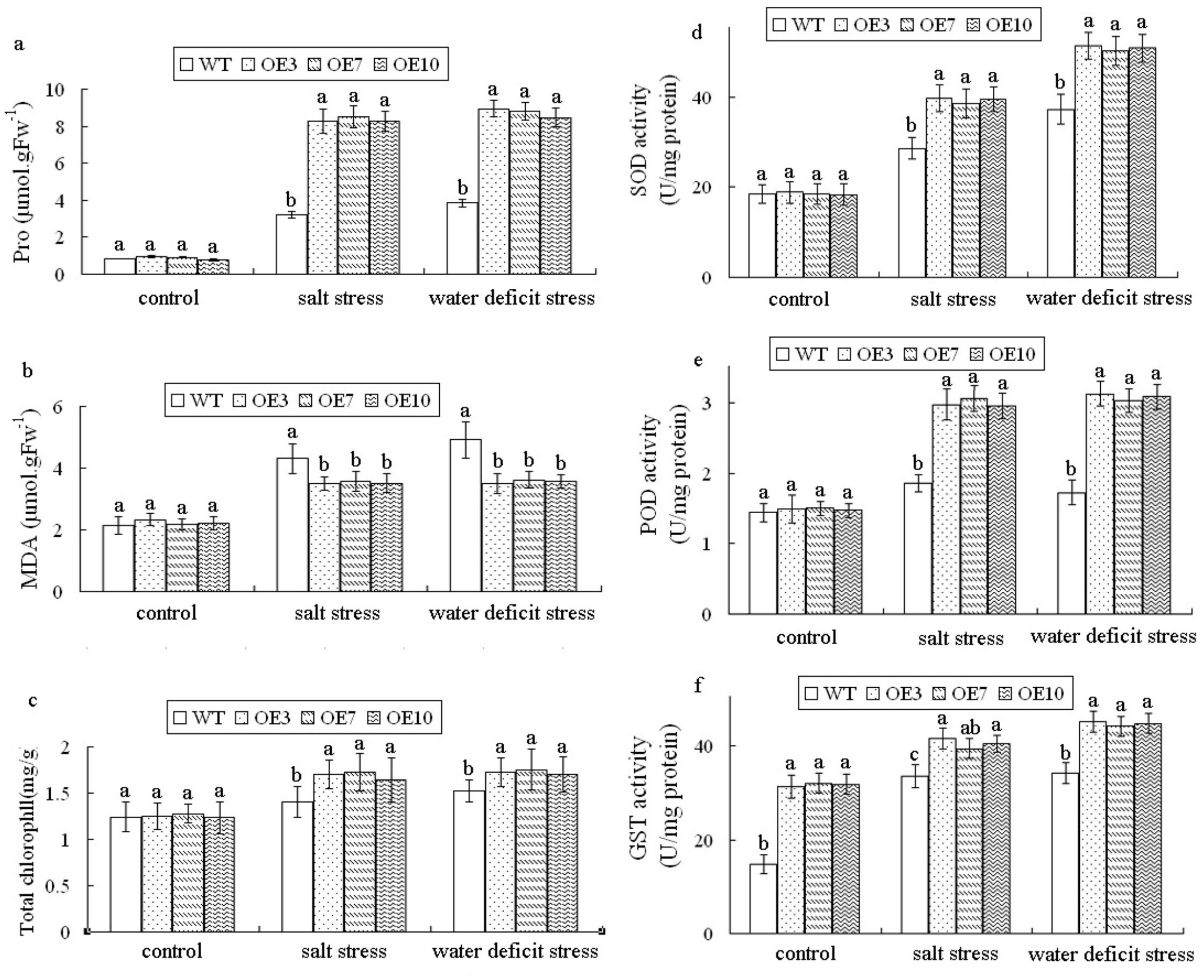
Changes in the content of proline, MDA, total chlorophyll content and the activities of SOD, POD and GST with different lines treated with NaCl (250mM) as salt stress or mannitol (300mM) as osmotic stress for 5d. a. proline content. b. MDA content. c. Total chlorophyll content. d. SOD activity. e. POD activity. f. GST activity. Values are mean±SD (n = 3), each from ten plants. The statistical significance was determined by Duncan’s multiple comparison tests. Different letters above bars indicate significant differences (P < 0.05) among different lines in the same treatment.

Proline, MDA and chlorophyll contents were analyzed (Fig 7A–7C) and the results showed that there were no difference between transgenic plants and WT. Under stress condition, proline and total chlorophyll contents increased in both transgenic plants and WT. However, the contents of proline and chlorophyll were significantly higher in transgenic plants than in WT plants during NaCl and mannitol stresses. The MDA content was also increased in both genotypes under stress condition, and WT showed significantly higher MDA content than that of transgenic plants, which means WT plants exhibited higher rates of cell damage than transgenic lines under stress conditions.

The activities of SOD and POD were also measured (Fig 7D and 7E) and the results showed no significant difference between transgenic and WT plants under normal conditions. Under stress conditions, the SOD and POD activity increased in both the transgenic and WT plants. However, SOD and POD activities in transgenic lines were significantly higher than in the WT plants during NaCl and mannitol stresses. Specifically, after 5d of NaCl stress treatment, the SOD activity levels of transgenic lines were 33% higher than that of WT, and the POD activity levels were 62% higher than in the WT. After 5d of mannitol treatment, the SOD and POD activity levels of transgenic plants were 34%, 73% higher than WT levels, respectively. The transgenic plants exhibited higher levels of GST activity during NaCl and mannitol stresses.

Taken together, these results suggest that *LeGSTU2* maintains cellular ROS homeostasis by scavenging ROS and preventing cell membrane damage. LeGSTU2 appears to play an important role in plant resistance to salt and drought stress.

## Discussion

Plants often encounter various environmental stresses, thereby generating reactive oxygen species (ROS), which likely cause membrane lipid peroxidation and yield highly cytotoxic products of oxidative DNA damage [5]. GST can be induced by diverse environmental stimuli. For instance, increased GST levels are used to maintain cell redox homeostasis and protect organisms against oxidative stress [14]. Although many GST genes have been cloned and analyzed, the role of a GST gene from tomato has been rarely investigated. In this study, *LeGSTU2*, a GST gene from tomato, was cloned and introduced to *Arabidopsis* to evaluation gene function during abiotic stress. Our results revealed that transgenic plant lines over-expressing the *LeGSTU2* gene improved drought and salinity tolerance of *Arabidopsis*.

Expression pattern analysis results revealed that *LeGSTU2* was highly expressed in roots and flowers; the transcript expression levels of *LeGSTU2* were induced by salt, osmotic and heat stress. A similar pattern was previously observed in *AtGSTU19*, (also named *AtGST8*, [27]), *OsGSTU3* and *OsGSTU4* [28]. The accumulation of *AtGSTU19* was induced by drought-associated oxidative stress [27], and its role in drought/oxidative tolerance was demonstrated in our recent findings. *OsGSTU3* and *OsGSTU4* are polyethylene glycol (PEG)-induced tau class GSTs, which were identified in rice roots. Salt stress, phytohormones, antioxidants and hydrogen peroxide (a strong oxidant) can rapidly induce *OsGSTU3* and *OsGSTU4* expression in rice roots. However, the roles of these two genes in stress tolerance have not been reported.

*LeGSTU2* in stress tolerance was functionally analyzed using the transgenic lines of *Arabidopsis*. Under normal conditions, transgenic plants showed no superiority in terms of germination and root growth to WT. However, when treated with NaCl or mannitol, transgenic plants showed higher germination rate and longer root growth than the WT, which suggests that *LeGSTU2* may play a role in the tolerance phenotype. Previous studies have shown that transgenic *parB* (*GST*) lines showed higher levels of root growth and relative biomass than Ler-0 when exposed to 100mM Na stress, which indicates that such phenotypes are derived from the expression of *parB* [29]. A recent study on the characterization of *ThGSTZ1* from *Tamarix hispida* showed similar results to those described above [12]. Furthermore, transgenic plants displayed the tolerance to salt/drought stress and conserved water to a higher extent than WT plants (Figs 5 and 6); these results suggest that *LeGSTU2* may play a positive role in the tolerance to salt/drought stress.

ROS homeostasis is important in protecting the normal metabolism. Plants can regulate ROS levels through ROS scavenging enzymes, such as SOD, POD and GST [30]. Moreover, over-expression of some GST genes has been shown to improve oxidative stress tolerance levels in transgenic plants [31]. In this study, over-expression of *LeGSTU2* showed increased activities of SOD and POD under stress conditions. It has been reported that over-expression of a specific antioxidant gene could influence the expression of other antioxidant genes; indeed transgenic GST expressing seedlings were shown to have a higher APX activity and MDHAR activity compared to nontransformed seedlings [8]. Tobacco plants expressing the GST gene showed an increase in GR avtivity [32]. Our results suggested that LeGSTU2 may act as a stress regulator through increasing the activity of antioxidant enzymes to strengthen the ROS scavenging ability or maintain ROS homeostasis.

Proline, as a compatible solute, functions in defense to maintain turgor pressure against osmotic challenge caused by water deprivation or extreme salinity. Proline also protects plant cells against oxidative damage by quenching ^1^O_2_ and directly scavenging HO^.^ [33]. Proline accumulation is also positively correlated with drought/salinity tolerance [34–35]. Our experiments showed that transgenic plants accumulated more proline contents under salt and drought stress than WT (Fig 7), thus, stress tolerance of transgenic plants was enhanced. Water deficit and salinity often cause rapid and excessive accumulation of ROS in plant cells, subsequently resulting in lipid peroxidation and accumulation of MDA [36–37]. In our study, MDA accumulation occurred in transgenic plants under salt stress to a less extent than in WT plants, this result suggested that lipid peroxidation was decreased in *LeGSTU2* transgenic plants. Relatively stable chlorophyll content was also observed in transgenic plants, this result indicated that these plants can maintain photosynthesis better than WT under stress conditions. Photosynthesis, photorespiration and light signaling in defense responses are correlated with ROS regulation [38]. Far-red insensitive 219 (FIN219)-interacting protein 1 (FIP1), a tau class GST gene from *Arabidopsis*, can interact with FIN219 to regulate cell elongation and flowering in response to light [39]. *AtGSTU17*, anther tau class GST gene, is also regulated by multiple photoreceptors, particularly phytochrome A under all light conditions, *AtGSTU17* also participates in various aspects of seedling development [15]. In conclusion, *LeGSTU2* possibly plays a positive role in the tolerance to salt/drought stress in *Arabidopsis*. Further analyses, particularlly on the role of phytohormones, are awaited to elucidate the function and the regulatory mechanism of *LeGSTU2* in plant stress responses.

## References

[1] Jakab G, Ton J, Flors V, Zimmerli L, Metraux JP, Mauch-Mani B. Enhancing Arabidopsis salt and drought stress tolerance by chemical priming for its abscisic acid responses. Plant Physiol. 2005;139(1):267–74. Epub 2005/08/23. doi: pp.105.065698 [pii] 10.1104/pp.105.065698 16113213 PMC1203376

[2] Saijo Y, Hata S, Kyozuka J, Shimamoto K, Izui K. Over-expression of a single Ca2+-dependent protein kinase confers both cold and salt/drought tolerance on rice plants. Plant J. 2000;23(3):319–27. Epub 2000/08/06. doi: tpj787 [pii]. . 10929125 10.1046/j.1365-313x.2000.00787.x

[3] Zhu JK. Salt and drought stress signal transduction in plants. Annu Rev Plant Biol. 2002;53:247–73. Epub 2002/09/12. 10.1146/annurev.arplant.53.091401.143329 12221975 PMC3128348

[4] Cummins I, Dixon DP, Freitag-Pohl S, Skipsey M, Edwards R. Multiple roles for plant glutathione transferases in xenobiotic detoxification. Drug Metab Rev. 2011;43(2):266–80. Epub 2011/03/24. 10.3109/03602532.2011.552910 . 21425939

[5] Marrs KA. The Functions and Regulation of Glutathione S-Transferases in Plants. Annu Rev Plant Physiol Plant Mol Biol. 1996;47:127–58. Epub 1996/06/01. 10.1146/annurev.arplant.47.1.127 . 15012285

[6] Bartling D, Radzio R, Steiner U, Weiler EW. A glutathione S-transferase with glutathione-peroxidase activity from Arabidopsis thaliana. Molecular cloning and functional characterization. Eur J Biochem. 1993;216(2):579–86. Epub 1993/09/01. . 8375395 10.1111/j.1432-1033.1993.tb18177.x

[7] Cummins I, Cole DJ, Edwards R. A role for glutathione transferases functioning as glutathione peroxidases in resistance to multiple herbicides in black-grass. Plant J. 1999;18(3):285–92. Epub 1999/06/23. . 10377994 10.1046/j.1365-313x.1999.00452.x

[8] Roxas VP, Lodhi SA, Garrett DK, Mahan JR, Allen RD. Stress tolerance in transgenic tobacco seedlings that overexpress glutathione S-transferase/glutathione peroxidase. Plant Cell Physiol. 2000;41(11):1229–34. Epub 2000/11/28. . 11092907 10.1093/pcp/pcd051

[9] Dixon DP, Edwards R. Glutathione transferases. Arabidopsis Book. 2010;8:e0131 Epub 2010/01/01. 10.1199/tab.0131 22303257 PMC3244946

[10] Takesawa T, Ito M, Kanzaki H, Kameya N, Nakamura I. Over-expression of a glutathion S-transferase in transgenic rice enhances germination and growth at loe temperature. Mol Breed. 2002;9:93–101.

[11] Kumar S, Asif MH, Chakrabarty D, Tripathi RD, Dubey RS, Trivedi PK. Expression of a rice Lambda class of glutathione S-transferase, OsGSTL2, in Arabidopsis provides tolerance to heavy metal and other abiotic stresses. J Hazard Mater. 2013;248–249:228–37. Epub 2013/02/06. doi: S0304-3894(13)00009-5 [pii] 10.1016/j.jhazmat.2013.01.004 . 23380449

[12] Yang G, Wang Y, Xia D, Gao C, Wang C, Yang C. Overexpression of a GST gene (*ThGSTZ1*) from *Tamarix hispida* improves drought and salinity tolerance by enhancing the ablity to scavenge reacive oxygen species. Plant Cell Tiss Organ Cult. 2014;117:99–112. 10.1007/s11240-014-0424-5

[13] Yu T, Li YS, Chen XF, Hu J, Chang X, Zhu YG. Transgenic tobacco plants overexpressing cotton glutathione S-transferase (GST) show enhanced resistance to methyl viologen. J Plant Physiol. 2003;160(11):1305–11. Epub 2003/12/09. . 14658382 10.1078/0176-1617-01205

[14] Chen JH, Jiang HW, Hsieh EJ, Chen HY, Chien CT, Hsieh HL, et al Drought and salt stress tolerance of an Arabidopsis glutathione S-transferase U17 knockout mutant are attributed to the combined effect of glutathione and abscisic acid. Plant Physiol. 2012;158(1):340–51. Epub 2011/11/19. 10.1104/pp.111.181875 pp.111.181875 [pii]. 22095046 PMC3252094

[15] Jiang HW, Liu MJ, Chen IC, Huang CH, Chao LY, Hsieh HL. A glutathione S-transferase regulated by light and hormones participates in the modulation of Arabidopsis seedling development. Plant Physiol. 2010;154(4):1646–58. Epub 2010/10/12. doi: pp.110.159152 [pii] 10.1104/pp.110.159152 20935176 PMC2996023

[16] Jain M, Ghanashyam C, Bhattacharjee A. Comprehensive expression analysis suggests overlapping and specific roles of rice glutathione S-transferase genes during development and stress responses. BMC Genomics. 2010;11:73 Epub 2010/01/30. 10.1186/1471-2164-11-73 1471-2164-11-73 [pii]. 20109239 PMC2825235

[17] Hoque MA, Uraji M, Banu MN, Mori IC, Nakamura Y, Murata Y. The effects of methylglyoxal on glutathione S-transferase from Nicotiana tabacum. Biosci Biotechnol Biochem. 2010;74(10):2124–6. Epub 2010/10/15. doi: JST.JSTAGE/bbb/100393 [pii] 10.1271/bbb.100393 . 20944411

[18] Dalton DA, Boniface C, Turner Z, Lindahl A, Kim HJ, Jelinek L, et al Physiological roles of glutathione s-transferases in soybean root nodules. Plant Physiol. 2009;150(1):521–30. Epub 2009/03/13. doi: pp.109.136630 [pii] 10.1104/pp.109.136630 19279195 PMC2675717

[19] Sytykiewicz H. Expression patterns of Glutathione Transferase Gene (GstI) in maize seedlings under juglone-induced oxidative stress. Int J Mol Sci. 2011;12(11):7982–95. Epub 2011/12/17. 10.3390/ijms12117982 ijms-12-07982 [pii]. 22174645 PMC3233451

[20] Lan T, Yang ZL, Yang X, Liu YJ, Wang XR, Zeng QY. Extensive functional diversification of the Populus glutathione S-transferase supergene family. Plant Cell. 2009;21(12):3749–66. Epub 2009/12/10. doi: tpc.109.070219 [pii] 10.1105/tpc.109.070219 19996377 PMC2814494

[21] Chi Y, Cheng Y, Vanitha J, Kumar N, Ramamoorthy R, Ramachandran S, et al Expansion mechanisms and functional divergence of the glutathione s-transferase family in sorghum and other higher plants. DNA Res. 2011;18(1):1–16. Epub 2010/12/21. doi: dsq031 [pii] 10.1093/dnares/dsq031 21169340 PMC3041506

[22] Hu ZL, Deng L, Yan B, Pan Y, Luo M, Chen XQ, et al Silencing of the LeSGR1 gene in tomato inhibits chlorophyll degradation and exhibits a stay-green phenotype. Biologia Plantarum. 2011;55(1):27–34. PubMed Central PMCID: PMC3268380.

[23] Xu J, Tian YS, Peng RH, Xiong AS, Zhu B, Jin XF, et al AtCPK6, a functionally redundant and positive regulator involved in salt/drought stress tolerance in Arabidopsis. Planta. 2010;231(6):1251–60. Epub 2010/03/11. 10.1007/s00425-010-1122-0 . 20217124

[24] Zhang X, Henriques R, Lin SS, Niu QW, Chua NH. Agrobacterium-mediated transformation of Arabidopsis thaliana using the floral dip method. Nat Protoc. 2006;1(2):641–6. Epub 2007/04/05. doi: nprot.2006.97 [pii] 10.1038/nprot.2006.97 . 17406292

[25] Ji W, Zhu Y, Li Y, Yang L, Zhao X, Cai H, et al Over-expression of a glutathione S-transferase gene, GsGST, from wild soybean (Glycine soja) enhances drought and salt tolerance in transgenic tobacco. Biotechnol Lett. 2010;32(8):1173–9. Epub 2010/04/13. 10.1007/s10529-010-0269-x . 20383560

[26] Diao G, Wang Y, Wang C, Yang C. Cloning and functional characterization of a novel glutathion S-transferase gene from *Limonium Bicolor* . Plant Mol BIol Rep. 2011;29:77–87.

[27] Bianchi MW, Roux C, Vartanian N. Drought regulation of GST8, encoding the Arabidopsis homologue of ParC/Nt107 glutathione transferase/peroxidase. Physiol Plant. 2002;116(1):96–105. Epub 2002/09/05. doi: ppl1160112 [pii]. . 12207667 10.1034/j.1399-3054.2002.1160112.x

[28] Moons A. Osgstu3 and osgtu4, encoding tau class glutathione S-transferases, are heavy metal- and hypoxic stress-induced and differentially salt stress-responsive in rice roots. FEBS Lett. 2003;553(3):427–32. Epub 2003/10/24. doi: S0014579303010779 [pii]. . 14572664 10.1016/s0014-5793(03)01077-9

[29] Ezaki B, Gardner RC, Ezaki Y, Matsumoto H. Expression of aluminum-induced genes in transgenic arabidopsis plants can ameliorate aluminum stress and/or oxidative stress. Plant Physiol. 2000;122(3):657–65. Epub 2000/03/11. 10712528 10.1104/pp.122.3.657 PMC58900

[30] Jiang Y, Yang B, Harris NS, Deyholos MK. Comparative proteomic analysis of NaCl stress-responsive proteins in Arabidopsis roots. J Exp Bot. 2007;58(13):3591–607. Epub 2007/10/06. doi: erm207 [pii] 10.1093/jxb/erm207 . 17916636

[31] Mittler R. Oxidative stress, antioxidants and stress tolerance. Trends Plant Sci. 2002;7(9):405–10. Epub 2002/09/18. doi: S1360-1385(02)02312-9 [pii]. . 12234732 10.1016/s1360-1385(02)02312-9

[32] Le Martret B, Poage M, Shiel K, Nugent GD, Dix PJ. Tobacco chloroplast transformants expressing genes encoding dehydroascorbate reductase, glutathione reductase, and glutathione-S-transferase, exhibit altered anti-oxidant metabolism and improved abiotic stress tolerance. Plant Biotechnol J. 2011;9(6):661–73. Epub 2011/04/01. 10.1111/j.1467-7652.2011.00611.x . 21450042

[33] Matysik J, Bhalu B, Mohanty P. Molecular mechanisms of quenching of reactive oxygen species by proline under stress in plants. Current Science. 2002;82:525–32.

[34] Kishor PBK, Hong Z, Miao GH, Hu CAA, Verma DPS. Over-expression of [delta]-pyrroline-5-carboxylate synthetase increases proline production and confers osmotolerance in transgenic plants. Plant Physiol. 1995;108:1387–94. . 12228549 10.1104/pp.108.4.1387 PMC157516

[35] O’Regan BP, Cress WA, Van Staden J. Root growth, water relations, abscisic acid and proline levels of drought-resistant and drought-sensitive maize cultivars in response to water stress. S Afr J Bot. 1993;59:98–104.

[36] Bartels D. Targeting detoxification pathways: an efficient approach to obtain plants with multiple stress tolerance. Trends Plant Sci 2001;7:284–6. 10.1016/s1360-1385(01)01983-5 11435150

[37] Zhu JK. Plant salt tolerance. Trends Plant Sci. 2001;6(2):66–71. Epub 2001/02/15. doi: S1360-1385(00)01838-0 [pii]. . 11173290 10.1016/s1360-1385(00)01838-0

[38] Kangasjarvi S, Neukermans J, Li S, Aro EM, Noctor G. Photosynthesis, photorespiration, and light signalling in defence responses. J Exp Bot. 2012;63(4):1619–36. Epub 2012/01/28. doi: err402 [pii] 10.1093/jxb/err402 . 22282535

[39] Chen C, Gao M, Liu J, Zhu H. Fungal symbiosis in rice requires an ortholog of a legume common symbiosis gene encoding a Ca2+/calmodulin-dependent protein kinase. Plant Physiol. 2007;145(4):1619–28. Epub 2007/10/30. doi: pp.107.109876 [pii] 10.1104/pp.107.109876 17965173 PMC2151686

